# Association Between Degeneration of the Paraspinal Muscles and Nerve Root Compression in Lumbar Disc Herniation Patients Who Underwent Percutaneous Endoscopic Lumbar Discectomy

**DOI:** 10.1111/os.70319

**Published:** 2026-04-13

**Authors:** Siming Xian, Lei Yuan, Zekun Li, Jiutian Huang, Miao Yu, Weishi Li

**Affiliations:** ^1^ Department of Orthopedics Peking University Third Hospital Beijing China; ^2^ Beijing Key Laboratory of Advanced Bioadaptable Orthopedic Implants Beijing China; ^3^ Engineering Research Center of Bone and Joint Precision Medicine Beijing China; ^4^ Peking University Third Clinical College, Peking University Health Science Center Beijing China

**Keywords:** lumbar disc herniation, nerve root compression, paraspinal muscle degeneration, percutaneous endoscopic lumbar discectomy

## Abstract

**Objective:**

While paraspinal muscle degeneration is closely linked to lumbar disc herniation (LDH), the relationship between radicular compression and paraspinal muscle degeneration remains controversial, and evidence regarding the effect of percutaneous endoscopic lumbar discectomy (PELD) on paraspinal muscle degeneration is limited. This study aimed to investigate the correlation between radicular compression and paraspinal muscle degeneration in LDH patients and to evaluate postoperative changes in paraspinal muscles following PELD.

**Methods:**

A total of 185 patients with single‐level L4‐5 LDH complicated by unilateral nerve root compression who underwent PELD between January 2020 and January 2023 were retrospectively enrolled. Preoperative and postoperative L3–S1 T2‐weighted MRI scans were acquired. Paraspinal muscle cross‐sectional area (CSA) and fat infiltration (FI) were measured using ImageJ, and total CSA (TCSA) and functional CSA (FCSA) were calculated. Paired‐samples *t*‐tests for bilateral paraspinal muscle differences and pre‐ to postoperative parameter changes; Spearman correlation analysis for continuous variable correlations; independent‐samples *t*‐tests, chi‐square tests or one‐way ANOVA for intergroup comparisons. A two‐tailed *p* < 0.05 was considered statistically significant.

**Results:**

Of this cohort, 108 patients completed the 3‐month postoperative follow‐up, and 29 underwent longitudinal follow‐up for over 12 months. At the L3–5 level, the CSA on the compression side was lower than that on the non‐compression side. At the L5–S1 level, the CSA and FI on the compressed side were greater than those on the opposite side. After PELD surgery, the CSA of the paraspinal muscles significantly increased, and the FI significantly decreased on the compression side. The duration of lower extremity pain (LEP) was positively correlated with the FI of the multifidus (MF) and erector spinae (ES) at all three levels; moreover, on the compression side, the correlation coefficient increased with decreasing segment length.

**Conclusion:**

Degeneration of the multifidus and erector spinae muscles, especially the FI, is significantly associated with nerve root compression. Moreover, the duration of LEP is meaningful for assessing paraspinal muscle degeneration. Paraspinal muscle degeneration significantly improved after PELD, and longer preoperative LEP duration was associated with greater improvement in FI.

## Graphical abstract

Paraspinal muscle degeneration can have adverse effects on the function of patients with lumbar disc herniation and is associated with nerve root compression. The duration of LEP was positively related with FI of paraspinal muscles. The paraspinal muscle degeneration of LDH patients improved after PELD surgery.

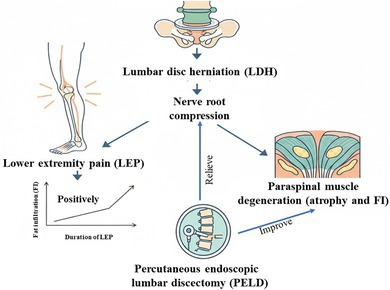

## 1 Background

Lumbar disc herniation (LDH) is a common clinical disease that can be observed at all ages. As an important cause of low back pain (LBP), it leads to a heavy economic burden and impairs quality of life [1, 2]. The paraspinal muscles mainly include the psoas major (PM), multifidus (MF), and erector spinae (ES) [3]. The MF and ES play major roles in the stabilization and mobility of the lumbar spine [4, 5]. In addition, the PM can help stabilize the lumbar spine, providing rigidity and stability to the lumbar spine without posterior support and serving as a compensatory mechanism for anterior pelvic tilt balance [3, 6, 7].

Previous studies have shown that degeneration of paraspinal muscles is related to age, sex, body mass index (BMI), and pain duration. Age, BMI, and pain duration can aggravate the degree of paraspinal muscle degeneration in females [8, 9, 10, 11]. Many studies have also highlighted that intervertebral disc degeneration is associated with degeneration of the paraspinal muscles, as indicated by increased fatty infiltration (FI), a decreased cross‐sectional area (CSA), and fibrous type transformation [12, 13, 14]. There are two main possible mechanisms by which LDH causes paraspinal muscle degeneration: nerve root compression can cause inflammation and a loss of neurotrophic effects, causing muscle damage and degeneration, and muscle atrophy can be caused by pain‐induced disuse [12, 15, 16]. However, the relationship between nerve root compression and paraspinal muscle degeneration is still controversial and is often neglected [2, 17, 18]. For example, degeneration of the paraspinal muscle is widespread, occurring not only on the compression side but also on the contralateral side and is not always limited to the compression level [8, 19].

With the evolution of minimally invasive endoscopic techniques, percutaneous endoscopic lumbar discectomy (PELD) has progressively become the predominant surgical approach for alleviating LDH‐related symptoms and restoring neurological function owing to its demonstrated advantages, including minimal tissue trauma, rapid postoperative recovery, and superior preservation of the paravertebral musculature [20, 21]. Nevertheless, the literature provides limited and inconclusive evidence regarding the improvement of paraspinal muscle degeneration in LDH patients with radicular symptoms following PELD surgery.

The primary objectives of this study were to (1) examine the relationship between radicular compression and associated paraspinal muscle degeneration in LDH patients and (2) assess postoperative changes in paraspinal muscles after PELD procedures.

## 2 Methods

### 2.1 Participants

The sample size was calculated using G*Power 3.1 software, with the primary outcome index being the change in paraspinal muscle FI after PELD. This study was approved by the Ethics Committee of Peking University Third Hospital (No. M2019488). A total of 185 patients with single‐level L4‐5 LDH complicated by unilateral nerve root compression who underwent PELD between January 2020 and January 2023 were retrospectively enrolled. The inclusion criteria for patients were as follows: (1) had single‐level L4–5 disc herniation and (2) had unilateral nerve root compression (MRI diagnosis accompanied by unilateral radiating pain in the lower extremity). The exclusion criteria for patients were as follows: (1) had a diagnosis of other spinal diseases and (2) had a history of previous spinal surgery.

The inclusion criteria for follow‐up analysis were as follows: (1) symptomatic improvement, (2) absence of new radicular symptoms, and (3) no radiographic evidence of recurrent disc herniation or other spinal pathologies.

### 2.2 Clinical and Imaging Data

The baseline data included sex, age, and BMI. The visual analog scale (VAS) was used to evaluate the intensity of low back pain and lower limb pain [22]. The Oswestry Disability Index (ODI) and Japanese Orthopedic Association (JOA) score were used to evaluate patients' physical function [23, 24]. Other clinical data included the duration of low back pain and lower extremity pain (LEP). The degree of nerve root compression was classified into four grades with reference to the method described by Park et al. [25] (Table 1).

**TABLE 1:** The grade of nerve root compression.

Grade	Definition
0	No nerve root compression with the nerve root clearly visualized on imaging
I	The nerve root being abutted or contacted
II	Nerve root displacement or deformation
III	Definite nerve root compression or complete nonvisualization of the nerve root (flattened or non‐visualized)

**FIGURE 1:**
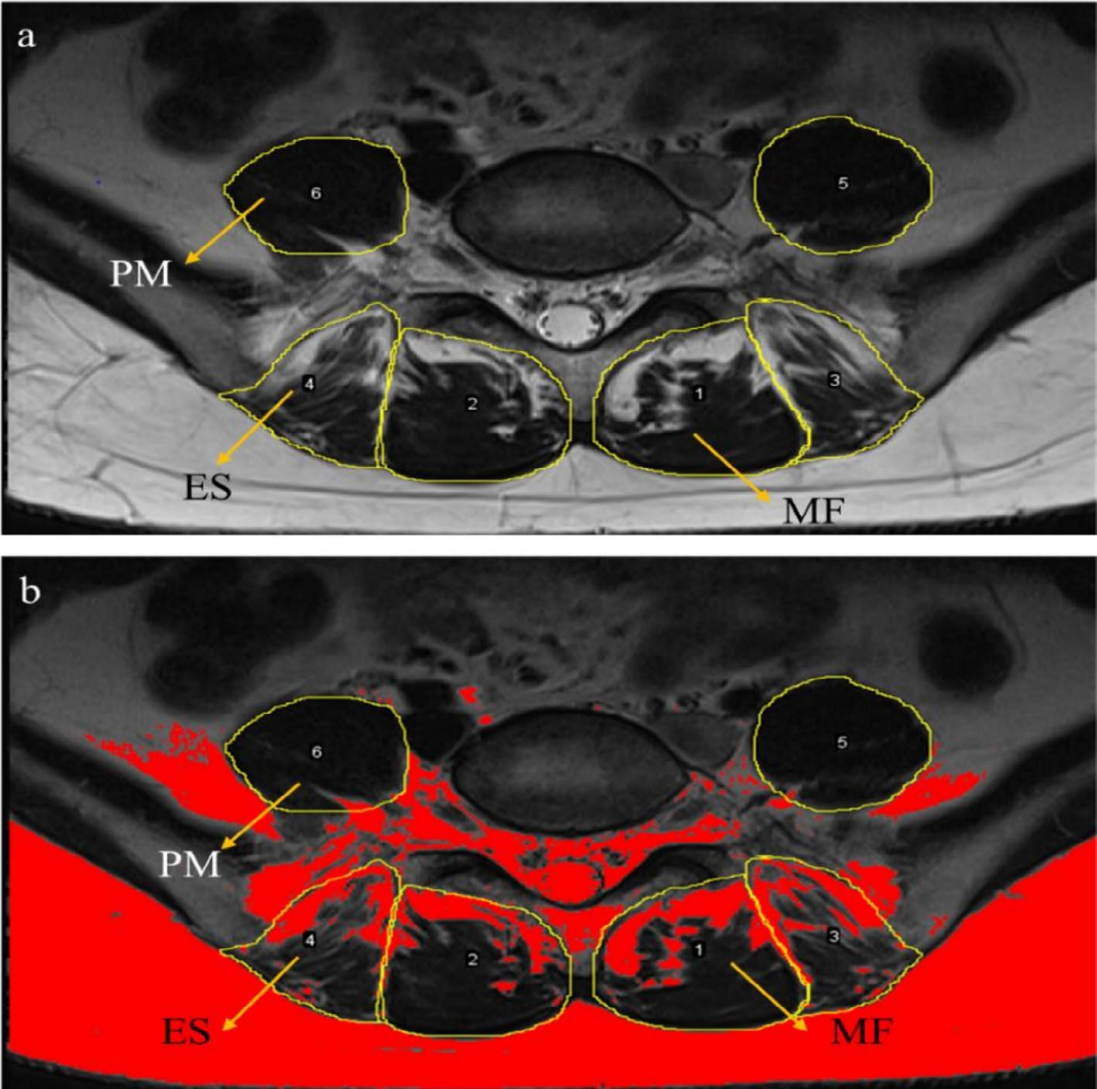
The CSA and FI of paraspinal muscles measured by ImageJ. ES, erector spinae muscle; MF, multifidus muscle; PM, psoas major muscle. (a) The contours of each paraspinal muscle were delineated, and the CSA was calculated via ImageJ according to the ruler. (b) Splitting and derivation of FIs via ImageJ. The automatic threshold setting of ImageJ software can distinguish between fat and muscle. Within the circles, red represents fat, and black represents muscle.

The CSA and FI of paraspinal muscles are used as indicators of muscle function [11, 26, 27]. T2‐weighted MRI scans of the L3–S1 segments (L3–4/L4–5/L5–S1) were acquired preoperatively, at 3 months postoperatively, and at the final follow‐up. The scan line was parallel to the disc and vertebral endplate. ImageJ was used to measure the CSA and FI of the MF, ES and PM on the compressed and uncompressed sides of the nerve root at each level (L3–4/L4–5/L5–S1) on T2 images, and the total CSA, TCSA = MF + ES, and functional CSA, FCSA = CSA*(1−FI) (TCSA) were calculated (Figure 1).

### 2.3 Statistical Analysis

IBM SPSS vn.27 software was used for the data analysis. Paired sample *t*‐tests were used to compare (1) bilateral paraspinal muscle differences at each level and (2) pre‐ versus postoperative changes in paraspinal muscle parameters.

Spearman correlation analysis was used to analyze correlations between continuous variables. Independent sample *T*‐tests, chi‐square tests, or one‐way ANOVA were used to compare differences between variables. A two‐tailed *p* < 0.05 was considered statistically significant.

## 3 Results

### 3.1 Demographic Data

The demographic and clinical characteristics of the participants are presented in Table 2. The study included 185 patients with LDH at L4–5 (116 males and 69 females), with an average age of 32.54 ± 11.53 years. Among the cohort, 108 patients completed 3‐month postoperative follow‐up assessments, whereas 29 patients maintained longitudinal follow‐up exceeding 12 months.

**TABLE 2:** Demographic and clinical characteristics of the patients.

	All	3‐month	> 12‐month
Age (years)	32.54 ± 11.53	35.57 ± 12.9	35.45 ± 12.59
BMI (kg/m^2^)	25.16 ± 3.93	25.02 ± 3.87	25.21 ± 3.52
Duration of LBP (months)	23.76 ± 40.97	—	—
Duration of LEP (months)	8.31 ± 14.04	—	—
VAS of LBP	4.28 ± 2.25	—	—
VAS of LEP	5.87 ± 1.83	—	—
ODI (%)	20.94 ± 8.58	—	—
JOA	15.72 ± 4.56	—	—

	*N* (%)	*N* (%)	*N* (%)
Male	116 (62.7)	63 (58.3)	16 (55.2)
Female	69 (37.3)	45 (41.7)	13 (44.8)
Degree of NRC			
I	45 (24.3)	27 (25)	8 (27.6)
II	80 (43.2)	53 (49.1)	15 (51.7)
III	60 (32.4)	28 (25.9)	6 (20.7)
The compression side			
Left	102 (55.1)	59 (54.6)	18 (62.1)
Right	83 (44.9)	49 (45.4)	11 (37.9)

Abbreviations: BMI, body mass index; JOA, Japanese Orthopedic Association; LBP, low back pain; LEP, lower extremity pain; NRC, nerve root compression; ODI, Oswestry Disability Index; VAS, visual analog scale.

The 185 patients had a mean duration of LBP of 23.76 months and a mean duration of LEP of 8.31 months. Nerve root compression occurred on the left side in 102 patients (55.1%) and on the right side in 83 patients (44.9%). Statistical analysis revealed no significant differences among the three patient groups in terms of sex, age, BMI, duration of LBP and LEP, laterality of nerve root compression, or severity of nerve root compression. The long‐term follow‐up subgroup (*n* = 29) had a mean follow‐up duration of 16.76 months.

### 3.2 Comparison Between the Paraspinal Muscles With and Without Nerve Root Compression

**FIGURE 2:**
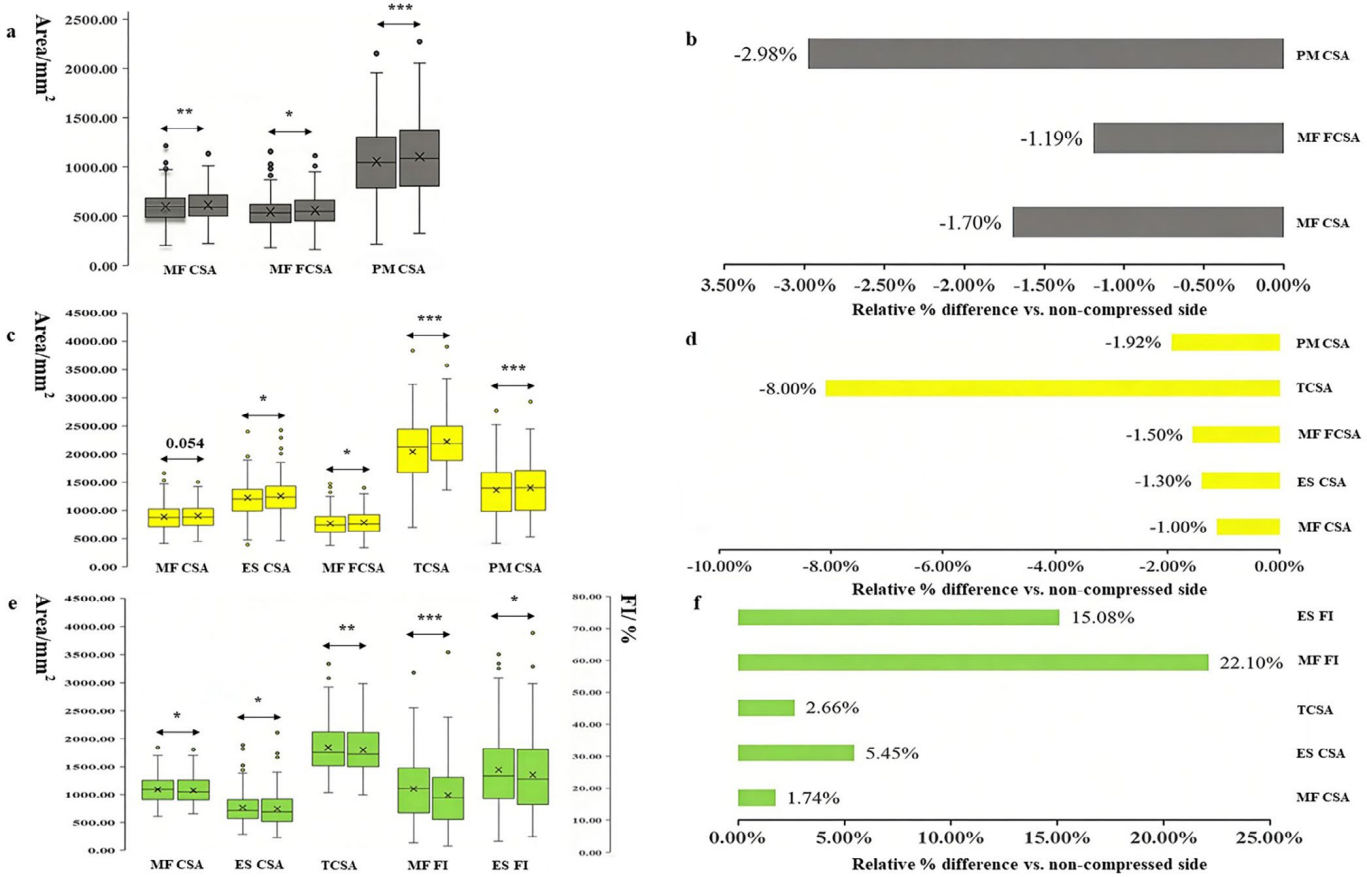
Comparison between the paraspinal muscles with and without nerve root compression. Box plots: Left = compressed side; (b, d, f) The percentage of the difference relative to the non‐compressed side. (a, b) L3–4, c‐d: L4–5, (e, f) L5–S1. CSA, cross‐sectional area; ES, erector spinae muscle; FCSA, functional cross‐sectional area; FI, fatty infiltration; MF, multifidus muscle; PM, psoas major muscle; TCSA, total cross‐sectional area; *p < 0.05, **p < 0.01, ***p < 0.001.

At the L3–5 levels, the CSA of the compression side was lower than that of the non‐compression side. At the L3–4 level, the CSA and FCSA of the MF and the CSA of the PM were significantly higher on the non‐compressed side (*p* = 0.003; *p* = 0.012; *p* < 0.001) (Figure 2a), and with the non‐compressed side as the reference, the relative percentage differences were −1.70%, −1.19%, and −2.98%, respectively (Figure 2b). At the L4–5 level, the CSA and FCSA of the MF, TCSA, and CSA of the ES and PM were significantly lower on the compression side (*p* = 0.054; *p* = 0.034; *p* < 0.001; *p* = 0.018; *p* < 0.001) (Figure 2c), and the relative percentage differences were −1.12%, −1.39%, −1.55%, −8.10%, and 1.92%, respectively (Figure 2d). Although the FI of the paraspinal muscles on the compressed side was consistently greater than that on the non‐compressed side at both L3–5 levels, these differences did not reach statistical significance.

In the L5–S1 segment, the study revealed significantly greater CSA and FI of the MF and ES on the compressed side than on the non‐compressed side (Figure 2e), whereas no significant interside differences were observed in the PM. Compared with the non‐compressed side, the relative percentage differences in CSA and TCSA of MF and ES were 1.74%, 5.45%, and 2.66%, respectively; additionally, the relative percentage differences in FI of MF and ES were markedly higher, reaching 22.10% and 15.08%, respectively (Figure 2f). In both the 3‐month follow‐up group and the group with follow‐up exceeding 12 months, the preoperative FI of the MF on the compressed side was significantly greater than that on the non‐compressed side.

Following grouping by the compressed side, no significant between‐group differences were observed in baseline characteristics or preoperative paraspinal muscle CSA and FI at any segment; in both groups, interside differences in paraspinal muscle CSA at and above the herniated segment were more pronounced than in the overall cohort, whereas differences in ES FI were minimal and clinically irrelevant, and conversely, at the L5–S1 segment, significant interside differences were predominantly noted in the FI of the ES and MF, with no marked differences in CSA.

### 3.3 Comparative Analysis of Preoperative vs. Postoperative Paraspinal Muscle Changes Following PELD

**FIGURE 3:**
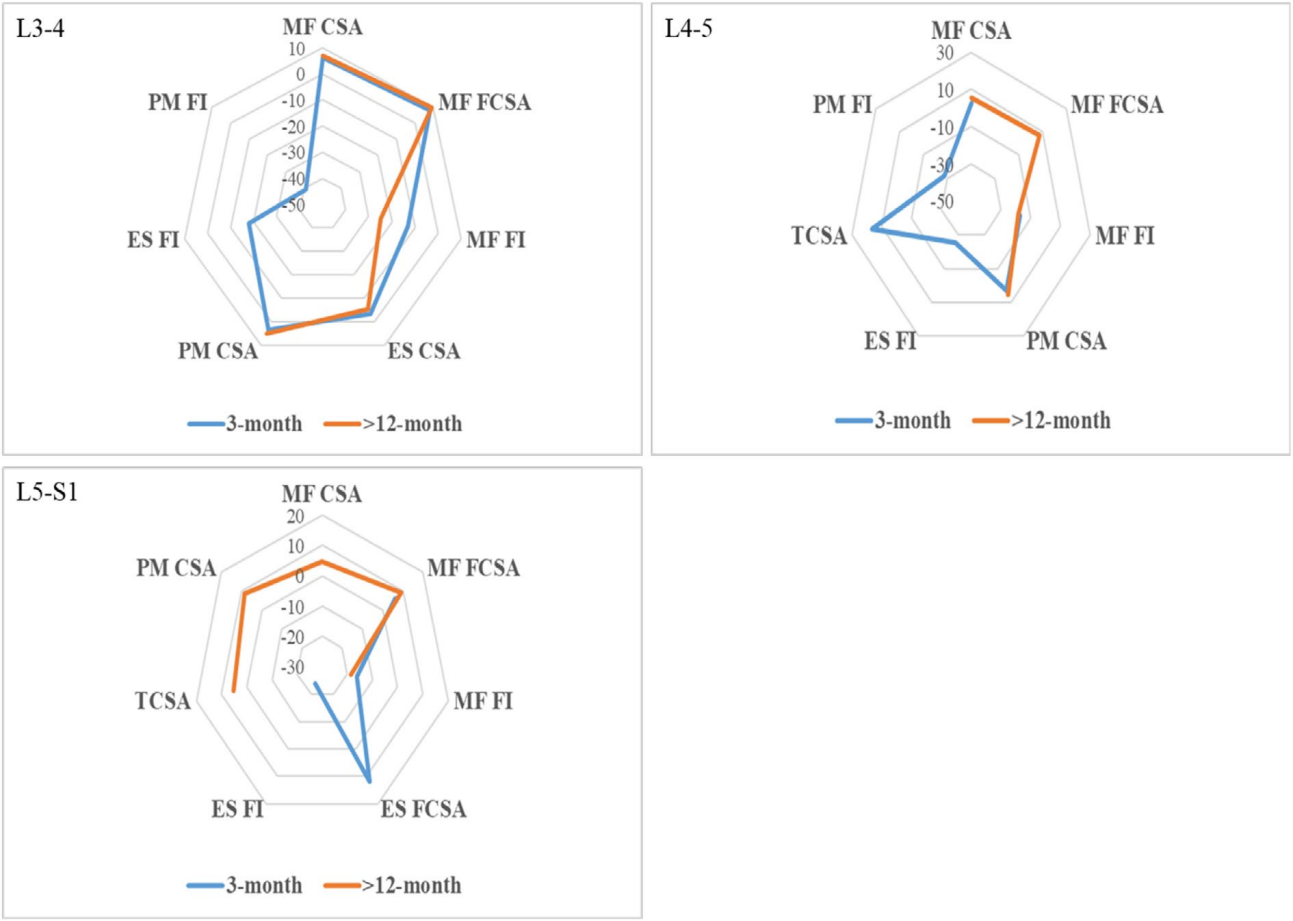
Comparison of paraspinal muscle indices before and after PELD. All values represent the percentage change relative to the preoperative baseline, with statistical significance. CSA, cross‐sectional area; ES, erector spinae muscle; FCSA, functional cross‐sectional area; FI, fatty infiltration; MF, multifidus muscle; PM, psoas major muscle; TCSA, total cross‐sectional area.

On the preoperative nerve root compression side, the CSA of the paraspinal muscles significantly increased at all three levels, and the FI of each paraspinal muscle was significantly lower than the preoperative value, regardless of whether the follow‐up was short‐term (3 months) or long‐term (> 12 months), with greater improvement in the long‐term follow‐up (Figure 3). On the non‐compressed side, such differences are not as pronounced. Moreover, no significant differences were observed in either the CSA or FI between the compressed and non‐compressed sides across all the spinal segments postoperatively.

### 3.4 Factors Associated With the CSA of Paraspinal Muscles in Each Segment

The correlation analysis revealed that age was negatively correlated with the CSA of the paraspinal muscles across all three segments, whereas BMI was positively correlated with the CSA of the paraspinal muscles. The duration of LEP was negatively correlated with the MF and ES on the compression side at L3–5. However, the duration of LBP and the degree of nerve root compression (NRC) were not significantly correlated with the CSA of the paraspinal muscles. Sex was correlated with the paraspinal muscle cross‐sectional area, with males tending to have larger CSA. After controlling for sex, age, and BMI, the durations of LBP and LEP did not significantly correlate with the CSA of paraspinal muscles, but LEP tended to be negatively correlated with this parameter.

The VAS scores for LBP and LEP were not significantly correlated with the CSA of the paraspinal muscles at each segment. The ODI and JOA scores were negatively correlated with the CSA and FCSA of ES at the L3–5 segments.

### 3.5 Factors Associated With FI of Paraspinal Muscles in Each Segment

Age, the severity of the NRC, and the duration of LBP or LEP did not significantly correlate with the FI of the paraspinal muscles. Moreover, BMI was positively correlated with the FI of the paraspinal muscles at all segments. The FI of the paraspinal muscles was lower in males than in females.

After controlling for sex, age and BMI, the duration of LBP was positively correlated with the FI of the MF at L3–5 but not with the FI of the paraspinal muscles at L5–S1. The duration of LEP was positively correlated with the FI of the MF and ES at all three levels, especially on the compressed side, where the correlation coefficient increased with decreasing segment length. The extent of the NRC was not significantly correlated with the FI of the paraspinal muscles (Table 3).

**TABLE 3:** Factors associated with FI of paraspinal muscles in each segment after controlling for sex, age, and BMI.

Segment		Side	Degree of NRC	Duration of LEP	Duration of LBP
L3–4	MF FI	Operated	0.025	**0.243** ^b^	**0.264** ^b^
		Opposite	0.083	**0.255** ^b^	**0.226** ^a^
	ES FI	Operated	0.108	**0.230** ^a^	**0.185** ^a^
		Opposite	0.16	**0.189** ^a^	0.116
	PM FI	Operated	0.011	0.046	−0.024
		Opposite	−0.152	−0.098	−0.088
L4–5	MF FI	Operated	0.025	**0.346** ^b^	**0.317** ^b^
		Opposite	0.075	**0.394** ^b^	**0.284** ^b^
	ES FI	Operated	−0.03	**0.209** ^a^	0.147
		Opposite	0.057	**0.235** ^b^	0.043
	PM FI	Operated	−0.066	0.000	0.118
		Opposite	−0.151	0.115	**0.183** ^a^
L5–S1	MF FI	Operated	−0.014	**0.407** ^b^	0.148
		Opposite	−0.004	**0.329** ^b^	0.146
	ES FI	Operated	0.066	**0.415** ^b^	0.067
		Opposite	0.111	**0.454** ^b^	0.047
	PM FI	Operated	0.026	0.001	−0.068
		Opposite	−0.167	0.094	−0.016

*Note*: Bold indicates the data are statistically significant.

Abbreviations: ES, erector spinae muscle; FI, fatty infiltration; LBP, low back pain; LEP, lower extremity pain; MF, multifidus muscle; NRC, nerve root compression; PM, psoas major muscle.

^a^ *p* < 0.05.

^b^ *p* < 0.01.

The VAS scores for LBP and LEP were not significantly correlated with the FI of the paraspinal muscles at any level. The ODI and JOA were positively correlated with the FI of the MF and ES at the L4–5 segment.

## 4 Discussion

In summary, radicular compression in LDH results in segment‐specific paraspinal muscle degeneration, characterized by decreased CSA at and above the herniated level, whereas fatty degeneration is more prominent at L5–S1. Correlation analysis indicated that the duration of LEP was significantly associated with FI of the paraspinal muscles. After PELD, the affected‐side paraspinal muscles significantly improve, with better recovery in the long‐term follow‐up, and interside differences disappear postoperatively.

### 4.1 Radicular Compression Is Associated With Paraspinal Muscle Degeneration

This study showed that at L5–S1, compressed‐side multifidus and erector spinae had significantly greater CSA and FI than the non‐compressed side, with notable interside FI differences when grouped by compression side; at superior levels, compressed‐side CSA was reduced, with no significant FI differences. These findings suggest that nerve root compression contributes to paraspinal muscle degeneration and notably induces an elevation in FI of the paraspinal muscles. In a study by Yazici and Yerlikaya [8], severe (> 50%) FI was associated with nerve root compression in patients with and without disc herniation and without radiculopathy, and the severity of FI increased with nerve root compression. Through a review and analysis of 67 relevant articles, Kalichman et al. [11] reported that nerve root compression can cause increased FI and that the lower the level of FI is, the more evident the infiltration is. Moreover, the present study demonstrated that the relative percentage differences in FI of the multifidus and erector spinae at the L5–S1 segment compared with the non‐compressed side were markedly higher than those in CSA and FCSA. This finding may explain why the increased CSA of these muscles on the compressed side at L5–S1 does not represent a true increase in muscular tissue area but instead results from compensatory fat infiltration replacing FCSA. This observation is consistent with the findings of previous studies [8, 11, 28]. Furthermore, muscle edema caused by nerve root compression may also be involved in the occurrence of this phenomenon [29].

Multiple previous studies have shown that a decrease in the multifidus muscle is associated with unilateral low back pain with or without nerve root compression, and this decrease is correlated with the duration of LBP [19, 30, 31, 32]. The results of the present study revealed a significant difference in the CSA in the multifidus muscle at and above the herniation level. As none of the appealing segments were root compressed, the decrease in the CSA of the multifidus was related mainly to the posterior ramus of the spinal nerves innervating the multifidus muscle, which can induce reflex inhibition mechanisms or pain‐induced disuse [19]. However, in some studies, no asymmetry of the multifidus muscle has been reported in patients with lumbar disc herniation with or without nerve root compression [8, 33].

There are few studies on the presence of the psoas major muscle. The present study revealed atrophy of the psoas muscle on the compression side compared with that on the non‐compression side at the level of herniation and above. Some studies have shown that atrophy of the psoas major muscle is associated with reduced activity due to pain and may be correlated with the duration and severity of pain (LBP and LEP) [32, 34, 35]. Given that the compressed side is typically the painful side and that, in this study, the duration of LEP tended to be negatively correlated with the CSA of the psoas major muscle, psoas atrophy may be related primarily to disuse due to pain. However, Kim et al. suggested that the CSA of the psoas major muscle is not related to LDH [30].

The current study demonstrated that the CSA of paraspinal muscles was related to BMI, sex, and age but not to the duration of LBP or LEP or the severity of nerve root compression. A higher BMI, younger age, and male sex tended to indicate greater CSA, which is consistent with the conclusions of previous studies [8, 9, 10, 11]. However, previous studies have shown that the factors associated with the CSA of paraspinal muscles are still controversial [8].

Previous studies have shown that FI of paraspinal muscles is associated mainly with age, sex, BMI, and pain duration, which is partially consistent with the findings of the present study [8, 10, 36]. The duration of LBP was positively correlated with the FI of the MF and ES at the L3–5 level. With a correlation greater than the duration of LBP, the duration of LEP was positively correlated with the FI of the MF and ES at all three levels; moreover, the lower the segment was, the greater the correlation coefficient was. These findings suggest that the duration of nerve root compression may be more meaningful for assessing paraspinal muscle degeneration.

### 4.2 PELD Can Effectively Ameliorate Paraspinal Muscle Degeneration

Previous studies have confirmed that patients with LDH demonstrate significant improvements in pain levels and functional scores following PELD. However, research on the effects of these procedures on paraspinal muscle degeneration is limited [20]. This study demonstrated that PELD leads to increased CSA and decreased FI in compressed‐side paraspinal muscles at both short‐term and long‐term follow‐ups. These findings indicate that PELD can effectively ameliorate paraspinal muscle degeneration with reliable long‐term efficacy. Numerous previous studies have indicated that pathological changes in the paraspinal muscles (e.g., asymmetric muscle fiber composition, hypertrophic adipocytes, and increased fat infiltration) are more pronounced in patients with LDH at segments inferior to the nerve root compression site, which may be associated with denervation of the paraspinal muscles [37, 38, 39]. The multifidus muscle exhibits the most significant changes, consistent with the findings of the present study [17].

PELD can effectively relieve nerve root compression, restore muscular innervation, potentially enhance muscle activity and nutritional supply, and reverse these pathological changes, ultimately improving paraspinal muscle atrophy and fat infiltration. This conclusion remains controversial, however, and awaits verification by long‐term postoperative pathological monitoring studies in the future [31]. Furthermore, relief of nerve root compression can alleviate patient pain, facilitate active muscle exercise, and ameliorate disuse muscle atrophy. The independent effects of surgical nerve root decompression and postoperative rehabilitation training on the paraspinal muscles could be further investigated in future research by implementing standardized preoperative and postoperative core stability training, combined with techniques such as isokinetic muscle strength testing and shear wave elastography.

This study revealed that the severity of LBP and LEP in patients with LDH was not related to the paraspinal muscles. However, the present study preliminarily confirmed that patients' functional scores (ODI and JOA) were correlated with paraspinal muscle degeneration at the herniation segments. In subsequent studies, a long‐term, longitudinal MRI database of paraspinal muscles can be established, artificial intelligence‐driven segmentation algorithms adopted to quantify the improvement rate of fat infiltration, and a causal relationship model formally constructed between fat infiltration and patients' functional scores.

In conclusion, this study clarifies the degenerative characteristics of paraspinal muscles related to radicular compression, identifies LEP duration as a key evaluation index, confirms the efficacy of PELD in improving muscle atrophy and FI, provides individualized rehabilitation guidance, fills the research gap on psoas major degeneration, and offers evidence for clinical practice and future studies. Meanwhile, postoperative rehabilitation is critical, including core stability training, paraspinal muscle strengthening, stretching, aerobic exercise, and posture correction, which help restore muscle strength, alleviate FI, and improve long‐term functional recovery.

## 5 Limitations and Strengths

This study has several strengths. We enrolled a well‐defined cohort with single‐level L4‐5 LDH and unilateral radicular compression, minimizing confounding factors. Detailed quantitative MRI analysis was performed to evaluate bilateral paraspinal muscle changes at multiple segments, and both short‐ and long‐term outcomes after PELD were assessed.

Several limitations should be acknowledged. It was a single‐center retrospective design with a relatively small long‐term follow‐up sample. The inclusion of only L4–5 single‐level disease may reduce generalizability. In addition, some significant CSA differences had limited clinical significance. Further large‐scale, multi‐center studies are warranted.

## 6 Conclusion

Paraspinal muscle degeneration can have adverse effects on the function of patients with LDH and is associated with nerve root compression. The duration of LEP is positively related to the FI of the paraspinal muscles. PELD can effectively ameliorate paraspinal muscle degeneration with reliable long‐term efficacy.

## Author Contributions

Administrative support: Miao Yu. Collection and assembly of data: Siming Xian, Lei Yuan, and Zekun Li. Data analysis and interpretation: Siming Xian, Lei Yuan, and Jiutian Huang. Manuscript writing: Siming Xian, Zekun Li, and Jiutian Huang. Scientific advice: Miao Yu and Weishi Li. Final approval of manuscript: All authors.

## Funding

This work was supported by China Disabled Persons’ Federation Fund of Assistive Technology (2021CDPFAT‐05), the Zhishan Foundation Huanghong & Lili Project 2022 and the Natural Science Foundation of Beijing Municipality (L232127).

## Ethics Statement

The study was approved by the “Ethics committee of Peking University Third Hospital.” The reference number was “no. M2019488.” All participants or their guardians provided written informed consent for participation and were free to withdraw from the study at any time.

## Consent

The authors have nothing to report.

## Conflicts of Interest

The authors declare no conflicts of interest.

## Supporting information


**Data S1:** Supporting Information.

## Data Availability

The data that support the findings of this study are available from Peking University Third Hospital. Restrictions apply to the availability of these data, which were used under license for this study. Data are available from the author(s) with the permission of Peking University Third Hospital.
